# Supplementing Coenzyme Q10 During the Vitrification and In Vitro Maturation of Dromedary Camel Oocytes Significantly Enhances Their Developmental Competence

**DOI:** 10.3390/ani16071079

**Published:** 2026-04-01

**Authors:** Karim A. Yaqout, Abou Bakr A. El-Wishy, Adel R. Moawad, Magdy R. Badr, Amr S. El-Shalofy

**Affiliations:** 1 Department of Theriogenology, Faculty of Veterinary Medicine, Cairo University, Giza 12211, Egypt; 2 Animal Science Program, College of Agriculture, Family Sciences, and Technology, Fort Valley State University, Fort Valley, GA 31030, USA; 3 Department of AI and ET, Animal Reproduction Research Institute, Agriculture Research Centre, Giza 12619, Egypt

**Keywords:** oocytes, dromedary camel, vitrification, IVM, coenzyme Q10, malondialdehyde, oxidative stress

## Abstract

This study aimed to evaluate the impact of coenzyme Q10 (CoQ10) supplementation during in vitro maturation (IVM) and/or vitrification of immature dromedary camel oocytes on their subsequent in vitro developmental competence. Additionally, total antioxidant capacity (TAC) and malondialdehyde (MDA) concentrations were assessed in the IVM spent medium. In experiment 1, cumulus–oocyte complexes (COCs, *n* = 808) collected from slaughtered dromedary camel ovaries were cultured in IVM media supplemented with either 0, 25, 50, or 100 μM CoQ10 for 36 h. Matured oocytes were then fertilized in vitro with epididymal camel spermatozoa. Eighteen hours post-insemination (pi), presumptive zygotes were cultured in vitro for 7 days. In experiment 2, a total of 875 COCs were randomly allocated to one of four experimental groups, namely (a) Vit−/IVM− (control) group, where COCs were vitrified in vitrification solution (VS; 25% DMSO + 25% EG) and matured in IVM media without CoQ10 supplementation; (b) Vit+/IVM− group, in which COCs were vitrified in a VS supplemented with 50 µM CoQ10 and matured in IVM media without CoQ10 supplementation; (c) Vit−/IVM+ group, where COCs were vitrified in VS without CoQ10 supplementation and matured in IVM media supplemented with 50 µM CoQ10; and (d) Vit+/IVM+ group, where COCs were vitrified in VS and matured in IVM media, both supplemented with 50 µM CoQ10. Following vitrification and warming, oocyte viability was evaluated morphologically and by trypan blue staining. Viable oocytes were then matured, fertilized, and cultured in vitro. In experiment 3, TAC and MDA concentrations in the IVM spent media were analyzed. Results showed that 50 µM CoQ10 supplementation to IVM media enhanced cumulus expansion, nuclear maturation, cleavage, and blastocyst rates (experiment 1). Adding 50 µM CoQ10 to the VS enhanced (*p* ≤ 0.05) oocyte viability compared to those vitrified in CoQ10-free media. Cumulus cell expansion and nuclear maturation rates were higher (*p* ≤ 0.05) in Vit−/IVM+ than in Vit+/IVM+ and Vit−/IVM− groups. Furthermore, cleavage and blastocyst rates were the highest (*p* ≤ 0.05) in Vit−/IVM+ group (experiment 2). The concentrations of TCA were higher, and the concentrations of MDA were lower (*p* ≤ 0.05) in Vit−/IVM+ than in other groups (experiment 3). In conclusion, supplementation of CoQ10 in the maturation medium of vitrified–warmed immature dromedary camel oocytes may enhance their in vitro developmental competence.

## Simple Summary

Cryopreservation of female gametes plays a key role in preserving fertility and maintaining the genetic diversity of animal populations. However, this process can cause significant damage to oocytes, reducing their developmental potential, an issue that is particularly challenging in camels, where assisted reproductive technologies are still developing. In this study, we examined whether adding the natural antioxidant coenzyme Q10 (CoQ10) during oocyte vitrification and in vitro maturation could help protect immature dromedary camel oocytes and improve their development after in vitro fertilization (IVF). Our results showed that supplementation with 50 µM CoQ10 enhanced oocyte viability, supported nuclear maturation, and increased the number of oocytes that developed into early embryos. CoQ10-treated oocytes also exhibited higher antioxidant capacity and reduced levels of harmful oxidative molecules. Overall, these findings indicate that CoQ10 effectively reduces oxidative damage and improves the quality and resilience of camel oocytes used in assisted reproduction. This approach has the potential to increase the success of embryo production in camels and contribute to the conservation and breeding of genetically valuable animals.

## 1. Introduction

Recent developments in assisted reproductive technologies (ARTs) have established cryopreservation as a reliable approach for the long-term storage of mammalian gametes, embryos, and gonadal tissues, thereby supporting fertility preservation and safeguarding valuable genetic resources [1]. Oocytes are highly susceptible to cryoinjury during freezing and thawing because they are large, single cells with a high lipid content. Consequently, they are prone to zona pellucida hardening, intracellular organelle damage, spindle abnormalities, DNA fragmentation, and unintended parthenogenetic activation. These alterations can adversely compromise the developmental competence of frozen–thawed oocytes [2].

Oxidative stress (OS) occurs when cellular antioxidant defenses fail to neutralize reactive oxygen species (ROS), resulting in an imbalance between the generation of ROS and the capacity of the cell to eliminate them. Because ROS are unstable and highly reactive, they readily interact with essential biomolecules—including proteins, lipids, and DNA—leading to oxidative damage and impaired cellular function [3]. Prolonged exposure to oxidative stress leads to depletion of the ovarian reserve, granulosa cell apoptosis, follicular atresia, chromosomal abnormalities, mitochondrial dysfunction, and a consequent decline in oocyte quality [4]. Vitrification has been shown to reduce oocyte viability and developmental competence by elevating reactive oxygen species (ROS) activity and increasing hydrogen peroxide (H_2_O_2_) concentrations [5,6]. Thus, incorporating antioxidants during the vitrification process may help enhance oocyte developmental competence while mitigating the oxidative stress induced by low temperatures [7].

Coenzyme Q10 (CoQ10) is a naturally occurring quinone located inside the inner mitochondrial membrane of all living organisms and plays a key role in electron transport within the mitochondrial respiratory chain. Beyond its essential function in cellular energy metabolism, CoQ10 also serves as a potent non-enzymatic antioxidant, capable of neutralizing ROS in both its oxidized (ubiquinone) and reduced (ubiquinol) forms [8,9]. The concentration of CoQ10 in human ovarian follicular fluid has been shown to correlate positively with oocyte and embryo quality, suggesting its potential role in folliculogenesis by supporting energy metabolism and protecting against oxidative stress [10]. CoQ10 concentrations decline with advancing female age, which may contribute to age-related reductions in fertility and the increased incidence of aneuploidy [11]. Therefore, CoQ10 supplementation has been recommended for women undergoing ART procedures to enhance oocyte and embryo quality [12]. In aged animal models, CoQ10 supplementation has been shown to delay ovarian-reserve depletion, restore mitochondrial gene expression in oocytes, and enhance mitochondrial activity [13]. CoQ10 supplementation has been shown to enhance the developmental competence of bovine oocytes during in vitro maturation (IVM) [14]. In addition, CoQ10 improved the survival and subsequent developmental rates of vitrified porcine oocytes [15].

Although live offspring have been produced through in vitro embryo production (IVEP) in dromedary camels, the efficiency of this technology still lags behind that of other domestic species [16]. In dromedary camels, reported cleavage rates range from 25 to 42%, and blastocyst rates rarely exceed 12% under standard IVEP conditions [17], underscoring the need for further optimization. Research on camel oocyte vitrification has largely centered on comparing freezing techniques and optimizing cryoprotectant type, concentration, and exposure duration [18]. However, despite these efforts, advancements in cryopreservation protocols have not yielded consistently satisfactory outcomes [19].

To the best of our knowledge, no studies have examined how CoQ10 supplementation during both in vitro maturation (IVM) and vitrification influences the preimplantation developmental competence of immature dromedary camel oocytes. We proposed that the addition of CoQ10 during these stages would support oocyte viability and enhance developmental outcomes. Accordingly, this study aimed to investigate the effects of CoQ10 supplementation during IVM and vitrification on the in vitro developmental potential of dromedary camel oocytes. In addition, oxidative status was assessed by measuring two critical indicators in the spent IVM medium: malondialdehyde (MDA), a stable end-product of lipid peroxidation widely used to quantify oxidative membrane damage, and total antioxidant capacity (TAC), which integrates the collective effect of enzymatic and non-enzymatic antioxidants and reflects the system’s ability to counteract ROS. Together, these measurements offer a comprehensive view of the redox environment during oocyte maturation.

## 2. Materials and Methods

All chemicals and reagents, unless indicated, were sourced from Sigma-Aldrich (St. Louis, MO, USA).

### 2.1. Ethics Statement

All experiments were conducted in accordance with the guidelines approved by Cairo University’s Institutional Animal Care and Use Committee (Vet CU12102021340).

### 2.2. Collection of Cumulus–Oocyte Complexes (COCs)

Dromedary camel ovaries were collected from a local slaughterhouse in Cairo, Egypt, between November 2021 and March 2022. Ovaries were transported in a thermos flask containing pre-warmed (30 °C), sterile normal saline solution (NSS; 0.9% NaCl). Cumulus–oocyte complexes (COCs) were aspirated from 2 to 8 mm follicles using a 20-gauge needle attached to a 20 mL syringe. The follicular fluid containing the COCs was transferred into 50 mL conical tubes filled with washing medium (HEPES-buffered TCM-199; H-TCM-199) supplemented with 10% (*v*/*v*) fetal calf serum (FCS). Tubes were kept at 39 °C for 10 min to allow the COCs to settle. After removing the supernatant, the remaining follicular fluid containing the COCs was poured into a 90 mm Petri dish. COCs exhibiting two to three layers of compact cumulus cells and a homogeneous, dark ooplasm were selected for subsequent experiments [20].

### 2.3. Vitrification and Warming Procedure

Selected COCs were first equilibrated for 3 min at 37 °C in an equilibration solution consisting of TCM-199 supplemented with 10% (*v*/*v*) FCS (base medium, BM), 12.5% (*v*/*v*) ethylene glycol (EG), and 12.5% (*v*/*v*) dimethyl sulfoxide (DMSO). Following equilibration, COCs were exposed for 60 s to a 100 µL drop of vitrification solution (VS) composed of 25% EG and 25% DMSO in BM and subsequently vitrified using the solid-surface vitrification (SSV) method as previously described [8]. Briefly, groups of five COCs in VS were aspirated into a glass pipette and expelled onto the dry surface of a hollow metal cube pre-cooled in liquid nitrogen (LN_2_). Once vitrified, droplets containing COCs were immediately transferred using LN_2_-cooled forceps into cryovials submerged in LN_2_. For warming, vitrified droplets were placed into 1 mL of warming solution (1 M trehalose in BM) for 3 min at 37 °C. COCs were then successively moved through drops of decreasing trehalose concentrations (0.5 M and 0.25 M in BM), followed by BM, for 3 min each at room temperature.

### 2.4. Assessment of COC Viability After Warming

The viability of COCs was assessed morphologically under a stereomicroscope immediately after warming. Oocytes displaying a spherical and symmetrical shape without signs of degeneration were classified as intact and selected for IVM, whereas those exhibiting cumulus cell loss, ruptured zona pellucida, fragmented cytoplasm, or other degenerative changes were classified as non-intact [21]. To confirm oocyte viability, a subset of morphologically intact COCs was subjected to trypan blue exclusion staining following the previously described method [22]. A 0.05% trypan blue solution was prepared by dissolving trypan blue powder in phosphate-buffered saline (PBS; pH 7.0), and staining was performed at room temperature for 2 min. Denuded COCs were then examined under a phase-contrast microscope: oocytes that excluded the dye were considered viable, whereas those that partially or completely absorbed the stain were classified as non-viable.

### 2.5. IVM Procedures and Evaluation of Cumulus Expansion and Nuclear Maturation

In vitro maturation of dromedary camel COCs was performed as previously described [17,23]. Briefly, COCs were washed twice in washing medium and once in maturation medium (TCM-199 with Earle’s salts) supplemented with 10% FCS, 5 µg/mL FSH, 5 µg/mL LH, 1 µg/mL 17β-estradiol, 5 µg/mL EGF, 50 µg/mL sodium pyruvate, 2.6 mg/mL sodium bicarbonate, and 50 µg/mL gentamycin. Groups of 10–15 COCs were then cultured in 100 µL of pre-warmed maturation medium under mineral oil for 36 h at 39 °C in 5% CO_2_ in air. Following IVM, cumulus expansion was assessed under a stereomicroscope by evaluating the degree of cumulus cell loosening and dispersion. Nuclear maturation was evaluated using the aceto-orcein staining technique as previously described [24]. After denuding the oocytes by repeated gentle pipetting, groups of 10–15 oocytes were placed on a clean 18 × 18 mm glass slide and covered with a coverslip supported by four Vaseline spots at the corners. Slides were fixed in a 3:1 ethanol: acetic acid solution for 24 h. Fixed oocytes were stained with 1% orcein for 3 min and examined under a phase-contrast microscope to determine nuclear maturation. Oocytes exhibiting a metaphase-II (MII) chromatin configuration were classified as mature.

### 2.6. In Vitro Fertilization, Embryo Culture, and Assessment of Embryo Development

Matured oocytes were fertilized in vitro using epididymal spermatozoa as previously reported [23,25]. Briefly, testicles were collected from slaughtered mature dromedary camels and transported to the laboratory in normal saline at 37 °C. Upon arrival, the testes were washed twice with normal saline, and the epididymides were dissected using a sterile scalpel. To collect epididymal spermatozoa, a small incision was made in the body of the epididymis with a sterile scalpel, and a 20-gauge sterile needle attached to a 5 mL syringe filled with flushing medium (TCM-199 with Earle’s salts supplemented with 9 mg/mL bovine serum albumin [BSA], 10 mg/mL theophylline, and 50 µg/mL gentamycin) was inserted into the incision. The flushing medium was gently pushed toward the cauda epididymis while applying light digital pressure along the epididymal tubules. A second small incision was made in the cauda region, and the droplets of flushed spermatozoa were collected into a 90 mm Petri dish. The collected suspension was kept at 39 °C for 10 min under 5% CO_2_ before being transferred to a 15 mL centrifuge tube. After centrifugation and removal of the supernatant, the sperm pellet was resuspended in 1 mL of flushing medium. Following a 1 h swim-up incubation at 39 °C, sperm motility was evaluated. For IVF, mature oocytes were washed three times in IVF medium and inseminated with motile spermatozoa at a final concentration of 2 × 10^6^ sperm/mL. Oocytes and spermatozoa were co-incubated for 18 h at 39 °C in 5% CO_2_ in air. Eighteen hours post-insemination (pi), presumptive zygotes were washed three times in TCM-199 supplemented with 5% FCS to remove residual cumulus cells, followed by two washes in embryo culture medium (TCM-199 with Earle’s salts supplemented with 5% FCS, 10 µL/mL essential amino acids, 5 µL/mL non-essential amino acids, and 50 µg/mL gentamycin). Groups of five zygotes were then cultured in 50 µL drops of embryo culture medium under mineral oil at 39 °C in a humidified atmosphere of 5% CO_2_ in air for seven days (Day 0 = day of insemination). Cleavage and blastocyst formation rates were recorded at 48 h and on Day 7 pi, respectively [20,25].

### 2.7. Evaluation of Total Antioxidant Capacity (TAC) and Malondialdehyde (MDA) in the IVM Spent Medium

In Experiment 3, total antioxidant capacity (TAC) and malondialdehyde (MDA) concentrations were measured in the IVM spent medium 36 h post-IVM. TAC was determined using a commercial assay kit (Antioxidant Capacity Assay Kit, Bio-diagnostic, Giza, Egypt) following the procedure described previously [26]. This assay evaluates the capacity of the sample to inhibit the formation of thiobarbituric acid reactive substances (TBARS) from sodium benzoate under oxidative conditions generated by Fenton’s reaction. The reaction product was quantified spectrophotometrically at 532 nm. Lipid peroxidation was assessed by measuring MDA levels using a commercial kit (Malondialdehyde Assay Kit, Bio-diagnostic, Giza, Egypt), according to a previously established protocol [27]. In this method, MDA reacts with thiobarbituric acid in an acidic medium at 95 °C for 30 min to form an MDA–TBA_2_ adduct. The resulting pink chromogen was measured spectrophotometrically at 534 nm.

### 2.8. Experimental Design

All experiments were conducted within the same season. In Experiment 1, the effects of supplementing the IVM medium with 25, 50, or 100 µM CoQ10, as well as without CoQ10 (0 µM; control), were evaluated. A total of 808 COCs were used; among these, 158 oocytes were allocated for assessing cumulus cell expansion and nuclear maturation, while the remaining 650 COCs were used to determine cleavage and blastocyst rates following IVF and IVC. Based on the results of Experiment 1, 50 µM CoQ10 was selected for subsequent experiments. In Experiment 2, the effects of 50 µM CoQ10 supplementation during vitrification and/or IVM on the developmental competence of vitrified–warmed immature dromedary camel oocytes were examined. A total of 875 COCs were used; of these, 158 oocytes were evaluated for cumulus expansion and nuclear maturation, whereas 717 were assigned to IVF. These COCs were randomly distributed into four groups: (a) Vit−/IVM−, COCs vitrified in VS and matured in IVM medium without CoQ10 (control); (b) Vit+/IVM−, COCs vitrified in VS supplemented with CoQ10 and matured in CoQ10-free IVM medium; (c) Vit−/IVM+, COCs vitrified in VS without CoQ10 and matured in IVM medium supplemented with CoQ10; and (d) Vit+/IVM+, COCs vitrified in CoQ10-supplemented VS and matured in CoQ10-supplemented IVM medium. Post-warming viability, cumulus expansion, nuclear maturation, cleavage, and blastocyst rates were recorded following IVM, IVF, and IVC. In Experiment 3, the effects of 50 µM CoQ10 supplementation during vitrification and/or IVM on MDA and TAC concentrations in the IVM spent medium were assessed. Spent media were collected post-IVM from the four treatment groups (Vit−/IVM−, Vit+/IVM−, Vit−/IVM+, and Vit+/IVM+) and stored at −20 °C until analysis.

### 2.9. Statistical Analysis

For each experimental group, a minimum of three replicates was performed. Data were expressed as mean ± SEM and analyzed using one-way ANOVA followed by Least Significant Difference (LSD) post hoc testing. Statistical analyses were conducted using SPSS^®^ software version 26.0 (SPSS Inc., Chicago, IL, USA). Differences were considered statistically significant at *p* ≤ 0.05.

## 3. Results

### 3.1. Experiment 1: Effects of CoQ10 Supplementation During IVM on Cumulus Expansion and Nuclear Maturation of Dromedary Camel Oocytes

Supplementation of the IVM medium with 50 µM CoQ10 significantly (*p* ≤ 0.05) enhanced both cumulus cell expansion and nuclear maturation (percentage of oocytes reaching the MII stage) compared with the 25 µM, 100 µM, and control (0 µM) groups (Table 1). In contrast, supplementation with 100 µM CoQ10 had a detrimental effect on in vitro maturation, yielding lower maturation rates than those observed in the 25 µM and 50 µM CoQ10 groups.

### 3.2. Effects of CoQ10 Supplementation During IVM on Cleavage and Blastocyst Formation Rates of Dromedary Camel Oocytes Following IVF and IVC

As shown in Table 2, supplementation of the IVM medium with 50 µM CoQ10 significantly improved (*p* ≤ 0.05) both cleavage rates at 48 h pi and blastocyst rates at day 7 pi compared with oocytes supplemented with 25 or 100 µM CoQ10, as well as the unsupplemented control group. In contrast, supplementation with 100 µM CoQ10 significantly reduced (*p* ≤ 0.05) cleavage and blastocyst rates relative to the control and all other CoQ10-treated groups. Based on these outcomes, 50 µM CoQ10 was selected for use in the subsequent experiments.

### 3.3. Experiment 2: Effects of CoQ10 Supplementation During Vitrification and/or In Vitro Maturation (IVM) of Dromedary Camel Immature Oocytes on Oocyte Viability, Cumulus Cell Expansion, and Nuclear Maturation

No significant difference (*p* > 0.05) was observed in the proportion of morphologically intact oocytes between the group vitrified with 50 µM CoQ10 and the group vitrified without CoQ10 (80.4% vs. 78.3%). However, trypan blue staining revealed that the percentage of viable oocytes was significantly higher (*p* ≤ 0.05) in the CoQ10-supplemented group compared with the non-supplemented group (73.8% vs. 64.6%). Following IVM, the proportions of COCs exhibiting cumulus expansion were significantly higher (*p* ≤ 0.05) in both the Vit−/IVM+ and Vit+/IVM+ groups compared with the Vit+/IVM− and control Vit−/IVM− groups (79.6% and 76.7% vs. 63.6% and 61.7%, respectively). Similarly, nuclear maturation rates were significantly greater (*p* ≤ 0.05) in the Vit−/IVM+ and Vit+/IVM+ groups than in the Vit+/IVM− and control Vit−/IVM− groups (72.5% and 69.2% vs. 61.1% and 58.1%, respectively) (Table 3).

### 3.4. Impacts of CoQ10 Supplementation During Vitrification and/or IVM of Dromedary Camel Immature Oocytes on Preimplantation Embryo Development Following IVF and IVC

**Figure 1:**
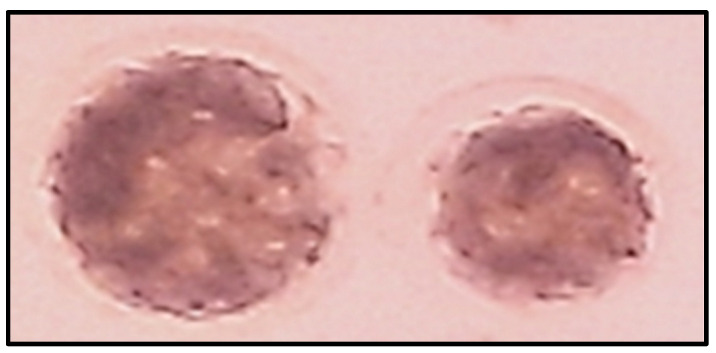
Day 7 post-insemination (pi) blastocyst dromedary camel embryos developed following in vitro maturation (IVM), in vitro fertilization (IVF), and subsequent in vitro culture (IVC) of vitrified/warmed germinal vesicle (GV)-stage cumulus oocyte complexes.

The effects of CoQ10 supplementation during vitrification and/or IVM on preimplantation embryo development of vitrified/warmed immature dromedary camel oocytes after IVM, IVF, and IVC are summarized in Table 4 and Figure 1. Cleavage rates at 48 h pi were significantly higher (*p* ≤ 0.05) in the Vit+/IVM− (34.5%), Vit−/IVM+ (41.9%), and Vit+/IVM+ (38.0%) groups compared with the Vit−/IVM− group (29.4%). Significant differences in cleavage rates were also observed among the Vit+/IVM−, Vit−/IVM+, and Vit+/IVM+ groups (*p* ≤ 0.05). Blastocyst (Figure 1) rates were higher (*p* ≤ 0.05) in the Vit−/IVM+ group (24.9%) than in the Vit+/IVM− (15.7%), Vit+/IVM+ (18.1%), and Vit−/IVM− (12.8%) groups. A similar pattern was observed when blastocyst rates were expressed as percentages of cleaved embryos (Table 4).

### 3.5. Experiment 3: Effects of CoQ10 Supplementation During Vitrification and/or IVM of Dromedary Camel Immature Oocytes on MDA and TAC Concentrations in IVM Spent Media

As shown in Table 5, the concentration of MDA in the IVM spent media was significantly lower (*p* ≤ 0.05) in the Vit−/IVM+ group compared with the Vit+/IVM−, Vit+/IVM+, and Vit−/IVM− groups (8.3 vs. 15.8, 16.2, and 17.9 nmol/mL, respectively). Conversely, TAC concentrations were significantly higher (*p* ≤ 0.05) in the Vit−/IVM+ group compared with the Vit+/IVM−, Vit+/IVM+, and Vit−/IVM− groups (18.9 vs. 8.7, 9.6, and 7.1 mmol/L, respectively).

## 4. Discussion

During in vitro manipulation, oocytes are exposed to multiple stressors that can induce oxidative stress through excessive ROS generation, which is known to negatively affect oocyte quality and subsequent embryonic development [18]. Furthermore, vitrification and warming have been demonstrated to compromise oocyte developmental competence due to thermal, mechanical, and chemical injuries that lead to membrane damage, altered cortical granule distribution, mitochondrial depolarization, and increased ROS production [28,29]. Because of their high lipid content, oocytes are particularly sensitive to oxidative stress; therefore, applying strategies that protect them from oxidative damage during in vitro handling is crucial [30]. In the present study, supplementation of 50 µM CoQ10 to the IVM medium significantly enhanced cumulus expansion, nuclear maturation, cleavage, and blastocyst rates (Experiment 1). Additionally, supplementation of 50 µM CoQ10 to the maturation medium of vitrified/warmed immature camel oocytes (Vit−/IVM+ group) markedly improved cumulus expansion, nuclear maturation, cleavage, blastocyst formation, and total antioxidant capacity (Experiments 2 and 3). These findings strongly support the hypothesis that inclusion of CoQ10 during in vitro handling of dromedary camel immature oocytes promotes their viability and developmental competence. Importantly, we observed that 50 µM CoQ10 in the maturation medium of dromedary camel immature oocytes significantly enhanced cumulus expansion and nuclear maturation compared with 25 and 100 µM CoQ10 and the control (0 µM). Previous work on bovine oocytes demonstrated that 40 µM CoQ10 improved ATP content and mitochondrial activity and reduced cell death relative to lower or higher concentrations [31]. Similarly, in porcine oocytes, 50 µM CoQ10 improved nuclear maturation in oocytes collected from both small and large antral follicles and particularly supported maturation in brilliant cresyl blue (BCB) negative COCs, suggesting a beneficial role for CoQ10 in low-quality oocytes [32]. These improvements may be attributed to enhanced mitochondrial function mediated by CoQ10 supplementation [31]. However, other studies have reported no improvements in nuclear maturation following CoQ10 supplementation (10–50 µM) [33], suggesting that discrepancies across studies may arise from variations in maturation media composition, culture conditions, or differences in oocyte stress levels.

Our data further demonstrated significantly higher cleavage and blastocyst rates in the 50 µM CoQ10 group. These findings align with previous work in pig oocytes, where 50 µM CoQ10 enhanced development to the blastocyst stage and increased blastocyst cell numbers [32]. Conversely, 100 µM CoQ10 supplementation in our study significantly reduced cleavage and blastocyst formation, consistent with earlier reports showing detrimental effects of high CoQ10 concentrations on porcine oocyte development [33]. It is worth noting that although the cleavage and blastocyst rates in the control groups were relatively low compared to those reported in cattle, they are consistent with current IVEP efficiencies in dromedary camels [16,17], underscoring species-specific challenges and the need for continued protocol optimization.

Oxidative stress is a major contributor to impaired developmental competence during IVEP and vitrification procedures, where mitochondrial dysfunction, reduced antioxidant capacity, and compromised meiotic integrity jeopardize embryo development [30]. CoQ10, a key electron transporter in the mitochondrial inner membrane, can mitigate these effects by enhancing energy production and reducing oxidative stress [34]. Our second experiment demonstrated that adding 50 µM CoQ10 to the vitrification medium of vitrified/warmed immature camel oocytes improved post-warming viability and developmental potential. Notably, the Vit−/IVM+ group (CoQ10 only during IVM) exhibited the highest improvement across all parameters, surpassing supplementation during vitrification alone (Vit+/IVM−), supplementation during both stages (Vit+/IVM+), and the unsupplemented control (Vit−/IVM−). This pattern suggests that vitrified/warmed oocytes experience substantial stress during the prolonged IVM period (36 h in camels) [20], and CoQ10 supplementation during this extended phase is critical for counteracting the detrimental effects of both vitrification and IVM. In contrast, the brief exposure to CoQ10 during vitrification—lasting only a few minutes—may not be sufficient for meaningful uptake or antioxidant action. Furthermore, under high-stress conditions, CoQ10 in vitrification solutions may exert a pro-oxidant effect or alter membrane properties in ways that impede subsequent uptake during IVM. Thus, the beneficial effects of CoQ10 appear to be exerted primarily during the prolonged IVM period rather than the short vitrification phase. [35]. Supporting this interpretation, previous studies have shown improved blastocyst development when CoQ10 is supplemented during recovery culture after vitrification, as well as enhanced survival and preservation of cortical granules in vitrified/warmed bovine oocytes treated with 50 µM CoQ10 [7].

In Experiment 3, supplementation of 50 µM CoQ10 during IVM of vitrified/warmed immature oocytes (Vit−/IVM+ group) produced the highest TAC and lowest MDA concentrations in the spent media. These results are consistent with earlier findings showing that 50 µM CoQ10 reduces ROS levels, decreases early apoptosis in porcine oocytes [32], and improves oxidative status in vitrified preantral follicles in mice [36]. Collectively, these findings support the conclusion that CoQ10 mitigates oxidative damage associated with in vitro manipulation [34], thermal stress [14], and aging [37] likely through improved mitochondrial function, thereby enhancing oocyte quality and developmental competence [32].

## 5. Conclusions

In conclusion, the processes involved in oocyte handling and culture in vitro disturb the intrinsic oxidant–antioxidant balance, thereby increasing oxidative stress and impairing embryo developmental potential. The addition of CoQ10 counteracts these detrimental effects by enhancing total antioxidant capacity and decreasing lipid peroxidation, as indicated by lower MDA levels. Collectively, our results indicate that CoQ10 supplementation during IVEP and/or vitrification enhances the viability and developmental competence of dromedary camel oocytes. Among the tested concentrations, 50 µM CoQ10 was the most effective and yielded the highest developmental indicators. Accordingly, we recommend the use of 50 µM CoQ10 during IVM of vitrified/warmed immature camel oocytes (Vit−/IVM+).

## Figures and Tables

**Table 1:** Influence of varying coenzyme Q10 (CoQ10) concentrations in the IVM medium on maturation outcomes of immature dromedary camel COCs.

CoQ10 (µM)	Total Number of COCs	Cumulus Cell Expansion n (% ± SEM)	MII n (% ± SEM)
25	192	134/192 (69.8 ± 1.2) ^a^	20/32 (62.5 ± 1.7) ^a^
50	204	163/204 (79.9 ± 0.9) ^b^	31/44 (70.5 ± 2.3) ^b^
100	189	111/189 (58.7 ± 1.4) ^c^	17/39 (43.6 ± 2.6) ^c^
0 (control)	223	153/223 (68.6 ± 1.6) ^a^	26/43 (60.4 ± 1.9) ^a^

MII: metaphase II. Values with different superscripts in the same column differ significantly (*p* ≤ 0.05).

**Table 2:** Effects of supplementing in vitro maturation (IVM) media with coenzyme Q10 (CoQ10) on cleavage and blastocyst rates following IVM, IVF, and IVC of dromedary camel immature oocytes.

CoQ10 (µM)	Cleavage 48 h-pi n (% ± SEM)	Blastocysts/Oocyte n (% ± SEM)	Blastocysts/Cleaved Embryos n (% ± SEM)
25	56/160 (35 ± 2.2) ^a^	33/160 (20.6 ± 1.5) ^a^	33/56 (58.9 ± 2.1) ^a^
50	71/160 (44.4 ± 2.3) ^b^	49/160 (30.6 ± 0.9) ^b^	49/71 (69.0 ± 1.7) ^b^
100	41/150 (27.3 ± 1.8) ^c^	20/150 (13.3 ± 1.8) ^c^	20/41 (48.8 ± 0.8) ^c^
0 (control)	62/180 (34.4 ± 1.4) ^a^	36/180 (20.0 ± 1.1) ^a^	36/62 (58.1 ± 1.3) ^a^

pi: post-insemination. Dissimilar superscripts within the same column indicate significant differences at *p* ≤ 0.05.

**Table 3:** Effects of coenzyme Q10 (CoQ10) supplementation during vitrification and/or in vitro maturation (IVM) on the maturation rates of dromedary camel immature COCs.

Groups	Total Number of COCs	Cumulus Cell Expansion n (% ± SEM)	MII n (% ± SEM)
Vit+/IVM−	214	136/214 (63.6 ± 2.3) ^a^	22/36 (61.1 ± 0.9) ^a^
Vit−/IVM+	221	176/221 (79.6 ± 2.1) ^b^	29/40 (72.5 ± 0.7) ^b^
Vit+/IVM+	210	161/210 (76.7 ± 2.6) ^b^	27/39 (69.2 ± 1.6) ^b^
Vit−/IVM−	230	142/230 (61.7 ± 1.5) ^a^	25/43 (58.1 ± 1.5) ^a^

Vit+/IVM−: CoQ10 (50 µM) supplementation during vitrification only. Vit−/IVM+: CoQ10 (50 µM) supplementation during in vitro maturation (IVM) only. Vit+/IVM+: CoQ10 (50 µM) supplementation during both vitrification and IVM. Vit−/IVM−: No CoQ10 supplementation during either vitrification or IVM. MII: metaphase II. Different superscripts within the same column indicate statistically significant differences at *p* ≤ 0.05.

**Table 4:** Impacts of coenzyme CoQ10 (CoQ10) supplementation during vitrification and/or IVM of dromedary camel immature COCs on cleavage and blastocyst rates following IVF and IVC.

Groups	Cleavage 48 h-pi n (% ± SEM)	Blastocysts/Oocyte n (% ± SEM)	Blastocysts/Cleaved Embryos n (% ± SEM)
Vit+/IVM−	61/178 (34.5 ± 1.3) ^a^	28/178 (15.7 ± 0.9) ^a^	30/61 (45.9 ± 2.3) ^a^
Vit−/IVM+	76/181 (41.9 ± 1.5) ^b^	45/181 (24.9 ± 1.3) ^b^	45/76 (59.2 ± 1.2) ^b^
Vit+/IVM+	65/171 (38 ± 1.1) ^ab^	31/171 (18.1 ± 1.6) ^ac^	31/65 (47.7 ± 1.9) ^a^
Vit−/IVM−	55/187 (29.4 ± 2.4) ^c^	24/187 (12.8 ± 2.8) ^ad^	24/55 (43.6 ± 1.7) ^a^

pi: post-insemination. Vit+/IVM−: only the vitrification medium was supplemented with 50 µM CoQ10. Vit−/IVM+: only the IVM medium was supplemented with 50 µM CoQ10. Vit+/IVM+: both vitrification and IVM media were supplemented with 50 µM CoQ10. Vit−/IVM−: no CoQ10 supplementation during either vitrification or IVM. Values with different superscripts in the same column indicate significant differences (*p* ≤ 0.05).

**Table 5:** Effects of CoQ10 supplementation during vitrification and/or IVM of immature dromedary camel oocytes on malondialdehyde (MDA) and total antioxidant capacity (TAC) concentrations in IVM spent media.

Groups	MDA (nmol/mL)	TAC (mmol/L)
Vit+/IVM−	15.8 ± 1.2 ^a^	8.7 ± 0.7 ^a^
Vit−/IVM+	8.3 ± 0.9 ^b^	18.9 ± 0.9 ^b^
Vit+/IVM+	16.2 ± 2.1 ^a^	9.6 ± 0.6 ^a^
Vit−/IVM−	17.9 ± 1.4 ^a^	7.1 ±1.1 ^a^

Vit+/IVM−: only the vitrification medium was supplemented with 50 µM CoQ10. Vit−/IVM+: only the IVM medium was supplemented with 50 µM CoQ10. Vit+/IVM+: both vitrification and IVM media were supplemented with 50 µM CoQ10. Vit−/IVM−: no CoQ10 supplementation during either vitrification or IVM. Values with different superscripts in the same column indicate significant differences (*p* ≤ 0.05).

## Data Availability

All generated data are provided in this submission.
